# An eye on hydration: efficacy of intraocular pressure to measure body water deficit

**DOI:** 10.1186/2046-7648-4-S1-A139

**Published:** 2015-09-14

**Authors:** Ian Stewart, Joseph Costello, Brittany Dias

**Affiliations:** 1 School of Exercise and Nutrition Sciences and Institute of Health and Biomedical Innovation, Queensland University of Technology, Brisbane, Australia; 2 Extreme Environments Laboratory, Department of Sport and Exercise Science, University of Portsmouth, Portsmouth, UK

## Introduction

Current best-practice hydration assessment include techniques involving isotope dilution to estimate total body water; osmolality of blood, saliva, or urine; specific gravity or colour of urine; and changes in body mass. These techniques are either prohibitively expensive, invasive, require clinical laboratory equipment, rely on a non-dehydrated baseline criterion, or on body fluids that are compromised in a dehydrated individual. In this study we report on the capability of intraocular pressure (the pressure within the eye; IOP), to assess dehydration. IOP can be measured quickly and accurately using a handheld device (tonometer), therefore offering portability, and sterility, and can be used by anyone following minimal training [1].

## Methods

Twelve healthy males (mean (SD): age 24 (2.3) yr, height 178 (6.1) cm, weight 75 (6.6) kg, VO_2max _56 (4.4) mL.kg^-1^.min^-1^, sum of eight skinfolds 75 (29) mm) completed two trials each comprising 150 minutes of treadmill walking (5 km,h^-1 ^and 1 % grade), in a hot and dry environment (40 °C and 20 % relative humidity). One trial was undertaken with fluid (water) replacement to minimise body mass changes (EUH) and one without fluid to maximise dehydration (DEH). The order of the trials was randomized and the trial days were separated by a minimum of seven days. At baseline and at 30 minute intervals participants were removed from the hot and humid environment into a temperate air-conditioned laboratory to have IOP, nude body mass and serum osmolality assessed.

## Discussion

IOP was progressively reduced during a period of exercise causing dehydration, but remained relatively stable if hydration was maintained. The large within and between individual variation in IOP meant that an absolute IOP value could not be identified to provide a criterion cut-off to represent a dehydrated condition. However utilising a change from a euhydrated baseline, delta IOP was significantly different between the conditions at increasing levels of body mass loss and serum osmolality.

## Conclusion

The evidence suggests that IOP is influenced by hydration status, likely due to the effect of a rise in blood osmolality on the rate of formation of aqueous humour.

**Table 1:** 

	Baseline	30	60	90	120	150
**IOP (mmHg)**						

EUH	14.4 (4.1)	15.5 (3.9)	14.7 (3.9)	14.1 (4.0)	14.5 (3.5)	14.2 (4.0)

DEH	15.6 (3.5)	14.2 (3.5)*	14.8 (4.1)	13.3 (3.3)	13.2 (3.6)*	13.0 (3.0)

**IOP delta (mmHg)**						

EUH		1.0 (1.7)	0.3 (2.7)	-0.3 (1.7)	0.0 (1.7)	-0.3 (2.4)

DEH		-1.5 (1.8)*	-0.8 (1.2)	-2.4 (1.7)*	-2.5 (1.6)*	-2.7 (1.9)*

**Body Mass delta (%)**						

EUH		0.0 (0.1)	-0.1 (0.1)	-0.1 (0.1)	-0.1 (0.2)	-0.2 (0.2)

DEH		-0.5 (0.1)*	-1.0 (0.1)*	-1.5 (0.1)*	-2.0 (0.2)*	-2.5 (0.2)*

**Serum Osmolality (mOsmol.kg^-1^)**						

EUH	291 (4.9)	291 (3.7)	291 (3.6)	291 (3.0)	292 (3.6)	292 (3.4)

DEH	292 (3.4)	293 (3.0)*	294 (2.7)*	297 (4.0)*	298 (4.5)*	299 (4.9)*

EUH, euhydrated trial; DEH, dehydrated trial. *Significantly different to EUH at same time point, p < 0.05.
